# Clinical applications of blood cell ratios in veterinary hematology: From diagnosis to prognosis

**DOI:** 10.1016/j.vas.2026.100622

**Published:** 2026-03-10

**Authors:** Narges Lotfalizadeh, Saeed Nazifi

**Affiliations:** Department of Clinical Sciences, School of Veterinary Medicine, Shiraz University, Shiraz, Iran

**Keywords:** Hematological ratios, Inflammation, Neutrophil-to-Lymphocyte Ratio (NLR), Platelet-to-Lymphocyte Ratio (PLR), Prognostic markers, Veterinary medicine

## Abstract

**Background:**

When examining routine blood tests, hematological ratios can demonstrate the systemic impact caused by diseases. Hematological ratios are less susceptible to interference than individual parameters.

**Objective:**

For a better understanding of these ratios, they must be collected and analyzed together in a comprehensive method. This review aims to categorize and discuss current hematological ratio studies in veterinary medicine, emphasize their potential, and identify future research needs.

**Methods:**

Studies investigating blood cell ratios in veterinary medicine were comprehensively searched in the literature utilizing PubMed, Web of Science, and Scopus databases, incorporating a variety of key words. All studies in which blood cell ratios were used in veterinary patients for diagnostic, prognostic, or monitoring purposes were included. Observational studies, retrospective studies, and experimental models were all combined in this review.

**Key findings:**

Neutrophil-to-lymphocyte ratio (NLR) is the most widely used ratio for diagnosing and prognosticating inflammatory, infectious, endocrine, cardiovascular, and neoplastic diseases in animals. The monocyte-to-lymphocyte ratio (MLR) and lymphocyte-to-monocyte ratio (LMR) are also relevant for immune-mediated, gastrointestinal, and neoplastic conditions. Animals have also been analyzed using ratios based on platelets (like the platelet-to-lymphocyte ratio (PLR) and red blood cells. Interest in the use of red cell distribution width (RDW) and platelet distribution width (PDW) in calculations has grown. When platelet activity and leukocyte responses are integrated into composite indices, they improve the prediction of disease severity.

**Conclusion:**

In veterinary medicine, these ratios are useful for detecting, monitoring, staging, and prognosing inflammatory conditions, including tumors, infections, autoimmune disorders, cardiovascular disease, and so on. The goal should be to minimize inconsistencies regarding the application of these ratios in the future while establishing a comprehensive standard framework.

## 1 Introduction

Hematological parameters are sensitive, effective, and essential measures of physiological and pathological alterations in humans and animals (Etim et al., 2014). Inflammation, hematological diseases, stress, and infection can be detected through blood indices derived from hematological parameters (Mekky et al., 2024). In addition, these parameters are easily obtainable when a hematology machine is available. Interactions between the white blood cells (WBCs), red blood cells (RBCs), and platelets play a crucial role in inflammatory and neoplastic conditions (Liu et al., 2024). The characteristics of individual cells, morphology, and specific indices, such as red cell distribution width (RDW) and platelet distribution width (PDW), provide insight into the mechanisms of disease. Elevated RDW and PDW may serve as hematological biomarkers to identify disease severity and progression (Martinez et al., 2019). There are various compensatory responses that can occur, including enhanced erythropoiesis or platelet production, as well as systemic inflammation or hypoxia in the tissues (García-Escobar et al., 2024). The ability to interpret hematological data, both diagnostic and prognostic, as well as develop targeted therapeutic approaches in veterinary and comparative medicine, depends on understanding these cellular dynamics (Becher et al., 2024).

The white blood cells play an integral role in defending the body from pathogens. There is, however, a possibility that a patient's white blood cell count may result in false negative results or may take a long time to reflect the progression of the inflammatory condition (Sindhu et al., 2025). There is growing evidence that hematological ratios such as NLR and PLR may serve as inflammation biomarkers (Sindhu et al., 2025; Gavazza et al., 2024). A hematological ratio can be an effective tool in veterinary oncology for providing veterinarians with information about disease outcomes (Gavazza et al., 2024). In contrast to these individual blood parameters, Hematological ratios are less susceptible to factors like age, sex, dehydration, and blood specimen handling, allowing a more accurate assessment of inflammation and immunity (Balta & Ozturk, 2015; Liu et al., 2022). Moreover, these indices are easy to measure and more stable than other inflammatory markers since they are derived from regular blood tests (Szydełko et al., 2021).

Hematological ratios can be used to demonstrate the systemic impact of diseases through routine blood tests. As a result, veterinarians and pet owners can communicate more easily and gain a better understanding of the importance of early diagnosis of illnesses in animals (Silva et al., 2024). Hematological examinations are minimally invasive procedures that veterinary clinicians perform before chemotherapy administration. The aggressive behavior of tumors makes it imperative to identify prognostic markers that can be used easily, repeatably, and inexpensively to monitor the course of the disease (Gavazza et al., 2024). Several studies have examined hematological parameters as potential prognostic indicators. There have been several studies in veterinary medicine that used hematological parameters to predict the outcome of a variety of diseases, including cardiovascular conditions (Jung & Kim, 2023; DeProspero et al., 2023; Mazzotta et al., 2016; Kocaturk et al., 2024), immune-mediated diseases (Duclos et al., 2025), infections and inflammation (Sindhu et al., 2025; Marchesi et al., 2024; Durán-Galea et al., 2024; Marzok et al., 2025; Engelbrecht et al., 2021; González-Domínguez et al., 2024; Çolakoğlu et al., 2025; Benvenuti et al., 2020), septic shock (Scalco et al., 2023a), hepatic disorders (Becher et al., 2024; Çolakoğlu et al., 2025), periodontal diseases (Silva et al., 2024), and parasitic diseases (Erdogan et al., 2025; Žvorc et al., 2010), along with cancers, namely, lymphomas (Gavazza et al., 2024; Sevim et al., 2024; Tagawa et al., 2021; Park et al., 2024), Nasal carcinomas and sarcomas (Rösch et al., 2024), soft tissue sarcomas (L Macfarlane et al., 2016), Ovarian tumors (Uğur et al., 2025; Çoban et al., 2021), Mammary gland tumors (Uribe-Querol et al., 2023), Oropharyngeal Tumors (Rejec et al., 2017), mast cell tumors (M Macfarlane et al., 2016), and others.

Compared with individual parameters, Hematological ratios yield results that are less susceptible to interference (L Macfarlane et al., 2016). Different parameters can therefore be considered for this purpose, such as leukocyte fractions. The neutrophil-to-lymphocyte ratio (NLR) has been studied in dogs with malignant and benign soft-tissue tumors, and NLR levels are significantly higher in malignant tumors (L Macfarlane et al., 2016). There have been studies evaluating Hematological ratios in many cases of systemic inflammatory diseases in cats and dogs (Sindhu et al., 2025; Donato et al., 2023). In addition to the NLR, platelet-to-lymphocyte ratio (PLR), platelet-to-neutrophil ratio (PNR), and monocyte-to-lymphocyte ratio (MLR), these biomarkers are considered cost-effective alternatives for diagnosing malignant tumors (Rejec et al., 2017). The interaction between platelets and lymphocytes contributes to cancer development. Malignant ovarian tumors, for instance, have a high PLR associated with an immunosuppressive microenvironment and a tendency to metastasize owing to tumor-induced thrombocytosis and lymphopenia (Raungkaewmanee et al., 2012).

In veterinary medicine, several Hematological ratios have been investigated individually, including the neutrophil-to-lymphocyte ratio (NLR), platelet-to-lymphocyte ratio (PLR), red cell distribution width/platelet ratio (RDW/PLT), and others. Yet no comprehensive review has collected all these indices and analyzed them clinically. Clinical and research teams need to collect and critically analyze these ratios together in order to better understand their diagnostic, prognostic, and monitoring potential. The present review aims to provide a comprehensive overview of the current studies in hematological ratios in veterinary medicine, emphasizing their utility and identifying future research needs.

## 2 Methods

### 2.1 Search strategy

Literature searches were conducted to identify studies that investigated blood cell ratios in veterinary medicine. Among them were NLR, PLR, MLR, and other hematological indices. Searches were conducted from October 2025 on electronic databases such as PubMed, Web of Science, and Scopus. It was possible to search using combinations of the following keywords: “Veterinary hematology,” “blood cell ratios,” “NLR,” “PLR,” “MLR,” “LMR,” “ELR,” “BLR,” “PLR,” “PNR,” “MPV/PLT,” “PDW/PLT,” “RDW/PLT,” “RDW/LYM,” “RDW/MPV,” “RDW/Ca,” “Hb/RDW,” “SII,” “SIRI,” “AISI,” “diagnosis,” and “prognosis” were included. Animal studies (dogs, cats, horses, and cattle) were included in the study. Comparative purposes and possible translational applications were discussed with reference to human studies.

### 2.2 Inclusion criteria

The studies were included if they reported the clinical application of blood cell ratios in veterinary patients, including diagnostic, prognostic, and monitoring applications. The main focus was on a combination of prospective and retrospective observational studies and experimental models. The background and discussion also included review articles, case reports, and studies with no quantitative data on blood cell ratios.

### 2.3 Data extraction

The extracted data were synthesized to assess the clinical relevance of each blood cell ratio in veterinary medicine. The criteria considered included: clinical utility for early diagnosis, correlation with disease severity, and prognostic significance for survival or treatment response. Human studies were included to identify gaps in veterinary research and potential translational applications.

## 3 Hematological ratios in veterinary medicine

### 3.1 Leukocyte-related ratios

#### 3.1.1 Neutrophil-to-lymphocyte ratio (NLR)

In veterinary medicine, measuring NLR is becoming increasingly popular, especially when it comes to assessing inflammation in dogs (Becher et al., 2021) and cats (Donato et al., 2023). As a biomarker derived from a complete blood count, NLR is both easy to access and cost-effective. It is used to evaluate the balance between inflammation and immunity (Salciccioli et al., 2015). A higher NLR is seen in dogs with dermatitis (Ferreira et al., 2021), pancreatitis (Neumann, 2021), immune-mediated anemia (Duclos et al., 2025), chronic enteropathy and inflammatory bowel disease (IBD) (Marchesi et al., 2024; Benvenuti et al., 2020; Becher et al., 2021), myxomatous mitral valve disease (Kocaturk et al., 2024; Tuna, 2024), septic inflammations (Hodgson et al., 2018), and meningoencephalitis (Park et al., 2022). Additionally, physiological stress and corticosteroids can result in an increase in NLR by increasing neutrophil concentrations and decreasing lymphocyte concentrations (Benvenuti et al., 2020; Huntley, 2022). Utilizing NLR alongside Animal Trauma Triage can also help assess trauma severity in animals quickly (Doulidis et al., 2024).

An increase in NLR in cats with non-septic effusions indicative of FIP could confirm systemic inflammation even in the absence of leukocytosis (Kopilović et al., 2023). A study compared the NLR between dogs with septic and non-septic systemic inflammation due to peritonitis and a control group of healthy dogs. The NLR ≥ 6 provided a sensitivity of 84.39 % and a specificity of 86.95 % in determining whether a dog has systemic inflammatory diseases (Hodgson et al., 2018). On the other hand, there was no significant association between NLR and the length of hospitalization, morbidity, or mortality associated with septic peritonitis (Hodgson et al., 2018). In cats and dogs with pancreatitis and prolonged recovery, NLR was significantly elevated (Neumann, 2021). When a dog has acute pancreatitis and a high NLR, the risk of death in the hospital increases, as does the duration of hospitalization (Johnson et al., 2023).

Among dogs with chronic enteritis, regardless of the cause, NLR increases significantly compared to control dogs (Marzok et al., 2025). In dogs with IBD, the NLR also provides diagnostic and prognostic information due to its distinct leukogram alterations (Marchesi et al., 2024). The presence of erosive or ulcerative intestinal lesions is also associated with neutrophilia, accompanied or unaccompanied by a left shift (Ridgway et al., 2001). There is evidence suggesting that an NLR of about 3.39 (2.34–5.81) is an independent risk factor for the diagnosis of IBD. Suspected cases showing IBD clinical signs, and NLR > 4.18 are considered high risk for severe IBD (Marchesi et al., 2024). Hemogram parameters were evaluated in dogs with cholangiohepatitis and cholangitis. NLR increases when neutrophilia or lymphopenia is present. Some cases of lymphopenia and neutrophilia have been observed in dogs with cholangiohepatitis or cholangitis. A significant difference was found between the patient and control groups when NLR rates were compared (Çolakoğlu et al., 2025). The NLR rate in Cholangitis or cholangiohepatitis complex patients is increased due to inflammation, and the increase in cortisone levels that arise during inflammation-induced stress. Neutrophil production may also be stimulated depending on inflammation severity, and apoptosis of lymphocytes affects NLR (Çolakoğlu et al., 2025; Neumann, 2021; Zahorec, 2001).

NLRs did not distinguish between dogs with liver portosystemic shunts who were either medically or surgically treated, or dogs with successful shunt surgery, or those with surgical complications. However, a lower NLR (<2.53) was correlated with dogs having only one portosystemic shunt closure procedure instead of two consecutive liver shunt operations (Becher et al., 2024). This means that evaluating the blood NLR may be useful in dogs who have liver shunts. The parameter may have little value in distinguishing dogs with liver shunts from those with other diseases, but it appears to be predictive of how many ligations are necessary to close the shunt (Becher et al., 2024).

NLR is correlated with indicators like C-reactive protein (CRP) in dogs with periodontitis (Silva et al., 2024; Rejec et al., 2017; Machado et al., 2021). Nonspecific inflammation is mediated by neutrophils, whereas adaptive immunity is stimulated by lymphocytes (Lu et al., 2021). However, the dogs with periodontitis showed the lowest neutrophil levels, significantly lower than both healthy and dogs with gingivitis (Silva et al., 2024). As adaptive immunity becomes more prominent during chronic inflammatory phases, this decrease may be attributed to an increase in lymphocytes (Kinane et al., 2000). The NLR < 2.577 may distinguish dogs with periodontitis from clinically healthy dogs (Silva et al., 2024).

Meningoencephalitis of unknown etiology (MUE) is characterized by higher NLR in dogs than in healthy dogs, according to a study (Park et al., 2022). A significant difference was also observed between dogs with MUE and those with idiopathic epilepsy (IE) and hydrocephalus. During the early phases of the disease, neutrophils play a crucial role in promoting inflammatory cell infiltration and contributing to lesion formation (Kanashiro et al., 2020; Steinbach et al., 2013). MRI and CSF analyses are needed for an antemortem diagnosis of MUE, which are expensive and require anesthesia, so the process can be frustrating for veterinarians as well as clients (Park et al., 2022). MUE dogs had significantly higher NLR than dogs with IE or hydrocephalus. Thus, the NLR could be used to distinguish dogs with MUE from dogs with IE or hydrocephalus. It can be used to determine whether an MRI is necessary to diagnose MUE or brain tumors (Šimundić, 2009). The optimal cutoff for the NLR is 4.16, which has a sensitivity of 71.1 % and specificity of 83.9 % when it comes to distinguishing MUE dogs from other forebrain conditions (Park et al., 2022).

There are various organs and tissues affected by Ehrlichiosis in dogs (Erdogan et al., 2025). The inflammation associated with ehrlichiosis has been associated with infectious and non-infectious causes, such as sepsis, malaria, babesiosis, trauma, and burns (Mylonakis et al., 2011; Sharma & Ismail, 2025). The leukocyte count of dogs with ehrlichiosis is different from the count in healthy dogs, and scientists have found that these dogs have fewer lymphocytes and more neutrophils (Bhadesiya & Raval, 2015). Ehrlichiosis causes a systemic inflammatory response syndrome in dogs, which increases NLR (Erdogan et al., 2025).

Both hyperadrenocorticism and hypoadrenocorticism can influence NLR. Dogs with adrenal insufficiency have a decreased NLR, and a combination of the Na/K ≤ 23 and the NLR ≤ 2.3 can be used reliably to diagnose hypoadrenocorticism (Zeugswetter & Schwendenwein, 2014). There is a high likelihood of finding NLR > 2.77 for hypercortisolism, with 100 % sensitivity and 63.64 % specificity (Yun et al., 2024). Therefore, NLRs can serve as supportive biomarkers for the evaluation of the adrenal gland in dogs, and they may be an appropriate tool for monitoring their responses to treatment (Yun et al., 2024).

In dogs with lymphocytic thyroiditis, which can cause hypothyroidism, NLR was significantly elevated than in healthy dogs (Elgalfy et al., 2025). Combined measurements of NLR and thyroglobulin autoantibodies can provide a comprehensive evaluation of autoimmune thyroiditis by indicating both specific immune activity and systemic inflammation (Elgalfy et al., 2025). There was a direct correlation between NLR and CRP, one of the best-established inflammatory markers that responds immediately to infection or inflammation (Malin & Witkowska-Piłaszewicz, 2022). A higher NLR in Lymphocytic thyroiditis dogs may be due to thyroid gland autoimmunity, or an immune response targeting thyroid tissue antigens, primarily thyroglobulin (Elgalfy et al., 2025; Sundberg, 2012).

A number of chronic diseases, including coronary heart disease, stroke, and cancer of solid organs (Zahorec, 2021), as well as diabetes, have been linked to NLR. There was a significant association between increases in NLR and impairment of neurological function early among diabetic and non-diabetic subjects (Vaitaitis et al., 2024; Fang et al., 2022). There was a substantial difference in NLR between dogs with diabetes mellitus and healthy controls. It was found that all dogs with diabetes mellitus had higher NLRs (Vaitaitis et al., 2024).

In cardiovascular pathological conditions, the NLR may increase due to physiologic stress and/or corticosteroid intake, resulting in increased neutrophils and reduced lymphocytes (Jung & Kim, 2023; Kocaturk et al., 2024). The observed increases in NLR may be related to neutrophils' immunoregulatory roles during the various stages of myxomatous mitral valve disease. By releasing inflammatory mediators, neutrophils contribute to the degeneration of the vascular wall and myocardial dysfunction, eventually leading to cardiac dysfunction (Jung & Kim, 2023; Kocaturk et al., 2024). A relationship was found between serum levels of cytokines, acute phase proteins, and antioxidants and their potential roles in the cellular, immune-mediated, and antioxidant mechanisms underlying the development and progression of myxomatous mitral valve disease in dogs (Kocaturk et al., 2024). NLR may serve as readily available markers for increased disease severity associated with increased diuretic dose and oxygen supplementation in dogs with cardiovascular diseases (DeProspero et al., 2023). High NLR was associated with worse short-term outcomes and higher mortality in hospital for dogs with chronic heart failure secondary to myxomatous mitral valve disease (DeProspero et al., 2023).

In cases of feline Hypertrophic Cardiomyopathy, heart failure, and arterial thromboembolism, NLR may serve as a prognostic factor (Fries et al., 2022; Esin & Uzun, 2025). Cats with cardiogenic arterial thromboembolism had the highest NLR values, followed by those with hypertrophic cardiomyopathy, both of which were significantly higher than those of healthy controls (Yoon & Li, 2025). There was a substantial increase in NLR in cats with congestive heart failure. Echocardiographic measures of left atrial size, left auricular function, thrombus formation, and edema were correlated with NLR measures (Fries et al., 2022; Yoon & Li, 2025). This indicates that NLR is associated with cardiac death risk (Fries et al., 2022; Esin & Uzun, 2025; Krofič Žel et al., 2025).

NLR has been reported to be significantly increased in canine and feline malignant tumors (Tagawa et al., 2021; Rösch et al., 2024; L Macfarlane et al., 2016). A unit increase in NLR has been reported to be associated with an increase in multicentric lymphoma progression and mortality, which is noteworthy. In this regard, elevated NLRs seem to be negatively associated with animal survival time (Park et al., 2024). Prognostic factors have been suggested for dogs with a diagnosis of large B-cell lymphoma based on PNR, NLR, and anemia levels. Higher PNRs above 0.032 were associated with earlier disease progression, and higher NLRs above 7.5 contributed to higher mortality rates after 180 days (Henriques et al., 2021). One study found that the NLR in lymphoma was twice as high as in the control group (5.50 versus 2.54) due to neutrophils produced during the inflammatory response and tumor-associated immunosuppressive mediators (Gavazza et al., 2024).

The median NLR varied significantly between high- and low-grade mast cell tumors in dogs as well as between tumors in different parts of the body (M Macfarlane et al., 2016; Skor et al., 2017). Based on a multivariate model, increasing NLR and age are associated with an increased risk of high-grade mast cell tumors (M Macfarlane et al., 2016). For predicting high-grade mast cell tumors, the NLR threshold value was 5.67 (sensitivity 86.7 %; specificity 54.2 %). Together with existing tools such as lesion appearance, location, etc., the NLR threshold of 5.67 may help predict mast cell tumor grade. If validated further, this biomarker may guide clinical decision-making prior to histopathological diagnosis (M Macfarlane et al., 2016). A significant increase in NLR was observed in dogs with oropharyngeal tumors when compared to healthy dogs, which could be indicative of systemic inflammation associated with the tumor (Rejec et al., 2017). Dogs have been diagnosed with a variety of ovarian tumors. Epithelial, germ-cell, and sex-cord stromal ovarian tumors are the three general categories of primary ovarian tumors (Terai et al., 2025). In dogs, epithelial tumors (adenomas and adenocarcinomas) and sex-cord stromal tumors (granulosa cell tumors) are the most prevalent (Manfredi et al., 2025). A dog with an ovarian tumor may develop anemia, neutrophilia, thrombocytosis, and lymphocytopenia (Uğur et al., 2025). Ovarian tumors in dogs can be predicted by NLR, an effective inflammatory marker (Uğur et al., 2025). In feline mammary carcinomas, NLR is considered a negative prognostic indicator and can be used to determine prognosis noninvasively before surgery (Petrucci et al., 2021).

#### 3.1.2 Monocyte-to-lymphocyte ratio (MLR)

Evaluations were also conducted on the MLR as a parameter designed to analyze differences between innate and adaptive immune cells (Vaitaitis et al., 2024). Generally, MLR is associated with cancer, but has also been studied in autoimmune disorders and cardiovascular diseases (Chen et al., 2019). Diabetes mellitus in dogs leads to notably higher MLRs than healthy dogs (Vaitaitis et al., 2024).

It was found that cats with increasing serum amyloid A (SAA) and globulin levels had higher MLRs than cats with lower albumin levels and decreased albumin-to-globulin ratios (AGR) (Donato et al., 2023). An elevated MLR was found in cats with left-shift neutrophils, toxic neutrophils, and reactive lymphocytes (Donato et al., 2023). Median MLR values for FIV-positive cats with systemic inflammatory response syndrome (SIRS) were significantly higher than those for healthy controls (Aksoy, 2025). Furthermore, an MLR > 0.27 was selected as the cutoff value for SIRS in cats, with 80 % sensitivity and 75 % specificity (Aksoy, 2025). When phagocytosis is required, as in infection and necrosis, animals exhibit reactive monocytosis (Weiss & Souza, 2010; Valenciano et al., 2010). Due to the small increase in monocytes in infectious inflammation, monocytosis occurs less frequently than neutrophilia. This explains why MLR correlations with inflammation are weaker than NLR correlations (Donato et al., 2023). For instance, canine periodontitis does not affect MLR (Silva et al., 2024).

There is evidence that MLR is a biomarker that could help predict the outcome of canine cardiovascular disorders, as well as the severity of myxomatous mitral valve disease, and there are negative effects of increased MLR on survival rates in dogs (Jung & Kim, 2023; Kocaturk et al., 2024). Moreover, MLR is related to increased risk of arterial thromboembolism in cats (Esin & Uzun, 2025).

Immune-mediated thrombocytopenia (IMHA) is characterized by systemic inflammation in dogs, and more pronounced regeneration may be expected when the disease and inflammation are more severe (Duclos et al., 2025). Since neutrophilia and monocytosis accompany regenerative responses and erythrophagocytosis, an increase in MLR is not surprising in IMHA. Dogs with IMHA showed enhanced MLR after cytokine-mediated nonspecific bone marrow stimulation (Duclos et al., 2025).

MLR has been evaluated as a diagnostic tool for some inflammatory conditions. Dogs with sepsis who had higher MLR values were at greater risk of death (Pierini et al., 2020). It was found that both MLR and monocyte counts were highly accurate in discriminating between dogs with IBD and clinically healthy dogs. Dogs with MLR > 0.14 are eight times more likely to have IBD (Marchesi et al., 2024). Biomarkers such as MLR have the potential to be used for assessing disease severity and monitoring (Marchesi et al., 2024). Despite the fact that an increased MLR rate correlates directly with disease severity and is associated with a poor prognosis for human liver diseases (Marchesi et al., 2024), dogs with primary cholangiohepatitis did not show any increase in MLR (Çolakoğlu et al., 2025).

Considering the chronic nature of neoplastic conditions, MLR measurements may be useful. Statistically significant differences were observed between subjects with Large B-cell lymphoma and healthy dogs for MLR (0.68 versus 0.13) (Gavazza et al., 2024). A high MLR could be explained by lymphopenia (35 %) and monocytosis (29 %) in dogs suffering from lymphoma (Gavazza et al., 2024). The MLR can be used as an adjunct to other tests to predict ovarian tumor progression in dogs (Uğur et al., 2025), in addition to metastasis (Catal et al., 2021).

#### 3.1.3 Lymphocyte-to-monocyte ratio (LMR)

The LMR represents a relatively new prognostic factor in the context of gastrointestinal malignancies and solid tumors in humans (Chan et al., 2017). The diagnostic and prognostic value of LMR in cats has been studied using comparisons of sick and healthy cats (Tsouloufi et al., 2021). Compared to healthy control cats, sick cats exhibited significantly lower LMRs, including those with infectious diseases, gastrointestinal disorders, cardiovascular complications, and Neoplasms. The LMR and serum glucose levels of sick cats were also negatively correlated. A notable feature is its potential application as a marker of systemic illness in cats (Tsouloufi et al., 2021).

Among dogs with multicentric large B-cell lymphoma, the LMR ≤ 1.2 was associated with a faster progression rate and shorter survival time (Marconato et al., 2015). According to another study on dogs with high -grade lymphoma, the optimal LMR cut-off value was 0.7 (Tagawa et al., 2019). Chemoimmunotherapy-treated dogs with lymphoma showed improved LMR as an independent prognostic indicator (Marconato et al., 2015). Study results on feline high-grade lymphoma showed that cats with a high LMR were more likely to live longer than those with a low LMR (Tagawa et al., 2021). Cats with LMR ≤ 3.4 had shorter Life Expectancy and shorter Time to Progression. There was an optimal cutoff value of 3.4 for cat lymphoma, higher than those reported in canine studies (Tagawa et al., 2021; Marconato et al., 2015; Tagawa et al., 2019), and this is logical since cats typically have more lymphocytes than dogs (Erdogan et al., 2025). In conjunction with other hematological indices, LMR is also a valuable predictor of outcome in dogs with cutaneous mast cell tumors (Skor et al., 2017). In contrast, LMR did not differ significantly between dogs with oral malignant melanoma and normal dogs (Murray & Blacklock, 2022), indicating that LMR did not affect survival, treatment response, or melanoma metastasis (Murray & Blacklock, 2022; Camerino et al., 2021).

#### 3.1.4 Eosinophil-to-lymphocyte ratio (ELR)

Human medicine is exploring the Eosinophil-to-Lymphocyte Ratio (ELR) as a hematological index that reflects the balance between inflammation mediated by eosinophils and immune regulation mediated by lymphocytes (Georgakopoulou et al., 2021). It serves as a simple, reliable biomarker for monitoring immune function and systemic inflammation (Urbańska et al., 2024) and has been associated with inflammation (Di et al., 2024; Zhang et al., 2025), severe allergic reactions (Selçuk & Keren, 2023), parasitic infections (Debrah et al., 2024), and certain types of cancer (Ohkuma et al., 2021; Ferro et al., 2019). A prognostic insight beyond absolute cell counts can be gained by integrating eosinophil functions into tissue infiltration, cytokine release, and modulation of inflammatory pathways. Research on ELR in veterinary medicine is minimal. Although dogs, cats, and horses have been reported to develop eosinophilia in response to parasitic, allergic, and neoplastic conditions (Guija-de-Arespacochaga et al., 2022; Lilliehöök et al., 2000; Center et al., 1990; Birkmann et al., 2024), no investigation into ELR has been reported. Neoplastic diseases, gastrointestinal disorders, endoparasites, respiratory diseases, neurological diseases, dermatological diseases, urogenital diseases, endocrine diseases, and many others were observed in dogs with marked eosinophilia (Guija-de-Arespacochaga et al., 2022). Lymphoma and mast cell tumor accounted for the most between tumors (Guija-de-Arespacochaga et al., 2022), as well as eosinophilic leukemia (Gilroy et al., 2011). Two other eosinophil-including indices, Neutrophil-to-eosinophil ratio (NER) and Relative eosinophil concentration (REC), were used in one study to predict cutaneous mast cell tumors in dogs (Skor et al., 2017). ELR may be useful in veterinary diagnostics for assessing disease severity, monitoring immune responses, and assessing prognoses.

#### 3.1.5 Basophil-to-lymphocyte ratio (BLR)

Chronic inflammation, hypersensitivity reactions, and tumor-associated immune modulation can be measured using the Basophil-to-Lymphocyte Ratio (BLR). It is possible to detect subtle alterations in systemic immunity using BLR because basophils play a key role in cytokine signaling and histamine release, despite their low number (Hadadi et al., 2022). Studies suggest that changes in BLR may correlate with disease progression, inflammation burden, and immune response in allergies and neoplasms (Urbańska et al., 2024; Hadadi et al., 2022; Cerci & Ucan, 2023). Currently, BLR research has not been conducted on veterinary species directly. Although dogs, cats, and horses exhibit basophilia along with hypersensitivity and hematologic disorders (Mau et al., 2025), clinical trials have not tested BLR as a ratio. The presence of basophilia was found as a paraneoplastic finding in cats suffering from alimentary T cell lymphoma (Balan et al., 2017; Strandberg et al., 2024) as well as dogs with chronic lymphocytic leukemia (Martínez-Caro et al., 2024) and basophilic leukemia (Azakami et al., 2019). BLR may serve as an accessible, novel biomarker for immune status, inflammation, and prognosis in veterinary medicine, thus bridging an important research gap.

### 3.2 Platelet-related ratios

#### 3.2.1 Platelet-to-lymphocyte ratio (PLR)

Combined with the functional roles of both cell types, the PLR gives insight into inflammation as well as coagulation (Xu et al., 2025a). Recent research indicates that this ratio may be an accurate biomarker for a variety of diseases, including cancer, inflammatory diseases, metabolic disorders, and neurodegenerative conditions (Suzuki et al., 2017; Gasparyan et al., 2019; Akboga et al., 2016; Frota et al., 2023). Both acute coronary syndromes and atrial fibrillation have been linked to the PLR as a significant indicator of adverse cardiovascular outcomes (Q Li et al., 2024; Li et al., 2017). By combining inflammatory and thrombotic reactions, PLR is capable of providing important information. Increased PLR indicates an inflammatory response, platelet activation, and a prothrombotic state (Li et al., 2017). Although PLR is less specific than markers like CRP, IL-6, and TNF-α in evaluating inflammation, thrombotic risk, and immune activation can be assessed with PLR efficiently and more cost-effectively, making it more convenient for clinical practice (Xu et al., 2025a).

The PLR is being investigated as a prospective biomarker in a variety of animal diseases. There was an increase in PLR in dogs that died of leptospirosis, suggesting it may be a negative prognostic factor (Durán-Galea et al., 2024). Similar data were found in canine parvovirus, in which dogs that died had a greater PLR than survivors, suggesting that PLR could be used as a prognostic tool, while MLR and NLR were less useful (González-Domínguez et al., 2024). Multiple organ dysfunction syndrome (MODS) may develop in dogs after systemic inflammatory response syndrome, depending on the severity and timing of medical intervention (Sindhu et al., 2025). PLR showed a significant decrease in MODS-affected dogs, contrasting with some prior reports, emphasizing how inflammatory markers vary across diseases (Sindhu et al., 2025).

There are several inflammatory conditions and immune-mediated diseases that can cause thrombocytosis in dogs (Woolcock et al., 2017). The PLR value of dogs and cats with pancreatitis was higher than that of healthy controls, although a correlation between disease intensity and PLR value was not found (Neumann, 2021). In such inflammatory conditions, PLR is likely to increase due to reactive thrombocytosis (Sindhu et al., 2025). Similarly, dogs affected by primary cholangiohepatitis displayed high NLR and PLR rates (Çolakoğlu et al., 2025). In dogs, the probability of developing IBD is four times higher for those with PLR > 131.6, according to a recent study (Marchesi et al., 2024). This can be explained by an increase in platelets in inflammatory conditions such as Chronic Enteritis, and by a decrease in lymphocytes in conditions such as IBD (Marchesi et al., 2024).

Studies on PLR have shown inconsistent results in periodontal disease. It was reported in one study that the PLR of dogs with gingivitis or periodontitis was lower than that of healthy controls, likely as a result of increased lymphocyte activity in response to chronic immune stimulation (Silva et al., 2024). PLR <81.492 was documented in dogs with gingivitis, and PLR <82.118 was calculated in dogs with periodontitis. The results of other studies, however, did not show a significant difference between healthy and affected dogs, suggesting the value of PLR may vary from study to study (Rejec et al., 2017).

There has also been research examining the ratio of platelets to lymphocytes in metabolic and endocrine disorders. Dogs with diabetes mellitus had significantly higher PLRs than healthy control dogs, reflecting alterations in platelet counts (Vaitaitis et al., 2024). Likewise, hypercortisolism was associated with significantly higher PLR than non-adrenal disease and healthy controls (Yun et al., 2024). As a support biomarker for hypercortisolism and assessing systemic inflammation, the PLR cut-off value of 285.0 distinguished hypercortisolism from non-adrenal illnesses with a sensitivity of 56.72 % and specificity of 70.69 % (Yun et al., 2024). Having high PLR levels negatively affects survival time in dogs with myxomatous mitral valve disease, according to analysis (Jung & Kim, 2023). As previously shown in veterinary studies, the platelets of myxomatous mitral valve disease patients were higher, and the lymphocytes were lower compared to healthy controls (Piantedosi et al., 2022; Cremer et al., 2014), and previous human studies also have shown a positive correlation between PLR and heart failure (Pourafkari et al., 2018).

The PLR is effective in predicting ovarian tumors in dogs, further highlighting its potential utility in treating various canine diseases (Uğur et al., 2025). PLR was associated with shorter overall survival and progression-free survival in human B-cell lymphoma patients (Wang et al., 2017). However, PLR indexes in large B-cell lymphoma dogs and healthy dogs failed to show statistically significant differences (120.57 versus 106.89) (Gavazza et al., 2024). The PLR value was found to be associated with overall survival, but not with the progression of diffuse large B-cell lymphoma in dogs (Henriques et al., 2021).

In dogs with malignant nasal tumors, PLR was significantly higher than in the control group; to detect malignant nasal cavity pathology, PLR ≥ 112.4 showed a sensitivity of 88 % and a specificity of 50 % (Rösch et al., 2024). PLR was also significantly higher in dogs with malignant nasal cavity conditions than in presumably benign ones. It was found that PLRs > 127.6 could detect malignant nasal cavity tumors with 76 % sensitivity and 57.1 % specificity compared to dogs with benign nasal cavity pathologies (Rösch et al., 2024). Aside from this, PLR was significantly greater in dogs with sarcomas and carcinomas than in the control group (Rösch et al., 2024). PLRs for healthy dogs without tumors in the oral cavity were 145, whereas PLRs of 290 indicated oral cavity cancer (Rejec et al., 2017). Generally, higher PLRs were observed in dogs with cancer, despite not being statistically significant, in line with another finding of a PLR of 250 in dogs with oral melanoma (Garcia et al., 2022).

#### 3.2.2 Platelet-to-neutrophil ratio (PNR)

The PNR is calculated from the absolute platelet value divided by the absolute neutrophil value. It is common for both humans and dogs with paraneoplastic hypercoagulability syndrome to experience changes in their PNR index as a result of neutrophils interacting with platelets in cancer (Gavazza et al., 2024). The presence of different mediators in hypercoagulability syndrome plays a crucial role in perpetuating inflammation and thus cancer progression (Henriques et al., 2021). In cancer, neutrophils and platelets can interact and cause changes in the PNR index, especially in dogs with paraneoplastic hypercoagulability syndrome. Hypercoagulability syndrome is perpetuated by several mediators that prolong inflammation and cancer progression (Henriques et al., 2021). There were significant differences between subjects with large B-cell lymphoma and healthy dogs when it came to PNR (25.89 versus 41.49)(8). Lymphoma patients presenting with PNRs below 0.032 have earlier disease progression, but there is no correlation between PNRs and lymphoma-specific survival (Gavazza et al., 2024). Furthermore, whole blood evaluations of dogs with diffuse B-cell small lymphocytic lymphoma found lower PNR values than those of healthy dogs (Sevim et al., 2025).

Dogs with gingivitis had a significantly lower and statistically significant PNR than dogs with no gingivitis. This may be due to the increased number of neutrophils (Silva et al., 2024). A dog with gingivitis and a healthy dog could be distinguished by the PNR when the ratio is lower than 34.49 (Silva et al., 2024).

Parvovirus-infected dogs with SIRS who died at first admission had significantly higher PNR values than dogs who survived (Sevim et al., 2025). In terms of sensitivity and specificity, PLR and PNR were the superior biomarkers for supporting a diagnosis of canine parvoviral enteritis and predicting disease outcomes (Sevim et al., 2025).

A recent prospective study compared the NLR and PNR in healthy cats and those with cardiovascular diseases, including hypertrophic cardiomyopathy and cardiogenic arterial thromboembolism, to determine their prognostic value (Yoon & Li, 2025). The results showed that cats with cardiogenic arterial thromboembolism had a significantly lower PNR than healthy cats. Despite no significant difference in PNR overall between normal and hypertrophic cardiomyopathy groups, cats with hypertrophic cardiomyopathy with PNR values below 40 had a significantly shorter median survival time and were nearly ten times more likely to die of cardiac disease (Yoon & Li, 2025). Compared to NLR and some echocardiographic parameters, PNR is more cost-effective, clinically relevant, and better at prognosticating feline hypertrophic cardiomyopathy (Yoon & Li, 2025).

#### 3.2.3 Mean platelet volume-to-platelet ratio (MPV/PLT)

A composite platelet index, MPV/PLT, reflects both platelet size (MPV) and circulating platelet mass (PLT), which is used to identify inflammatory or reactive changes in the hemostatic system. Human medicine has widely explored this ratio, but few studies have been conducted in veterinary medicine. Increasing MPV, which reflects platelet size, indicates an increase in platelet production and activation during chronic inflammation (Korniluk et al., 2019). A higher MPV was found in dogs infected with canine parvovirus compared with controls, indicating that platelet activation is present in dogs with parvoviral enteritis, potentially affecting the outcome of the disease (Engelbrecht et al., 2021).

In dogs with oral inflammatory disorders and tumors, MPV/PLT has been evaluated, but the results revealed only slight increases without clear diagnostic differentiation (Rejec et al., 2017). There was also limited responsiveness to chronic inflammation in MPV/PLT in a recent analysis of canine periodontal disease (Silva et al., 2024). Similarly, MPV/PLT demonstrates inconsistent diagnostic performance in systemic hepatobiliary inflammation. Dogs with primary cholangiohepatitis and cholangitis showed changes, but did not demonstrate significant differences between affected and healthy animals (Çolakoğlu et al., 2025). Further research into canine inflammatory conditions shows that MPV-based parameters are not consistently related to inflammatory burden (Kocaturk et al., 2024). There was, however, a significant difference between the ratios in cats with non-septic FIP effusions, and they were markedly higher, indicating disease- and species-specific variations (Kopilović et al., 2023).

#### 3.2.4 Platelet distribution width-to-platelet ratio (PDW/PLT)

An emerging hematological index that incorporates platelet size variability with platelet count is the Platelet Distribution Width-to-Platelet Ratio (PDW/PLT), indicating the presence of more heterogeneous platelets (Phillips et al., 2022). It may be used to identify inflammatory or pathological processes (Fonseca et al., 2023). There are limited direct studies of PDW/PLT in animals, but available research indicates that platelet indices alter with a variety of diseases, including pyometra in dogs (Demeli & Meyer, 2023), canine parvovirus enteritis (Engelbrecht et al., 2021), feline panleukopenia (Yanar, 2024), thrombocytopenia (Souza, 2022), periodontal disease (Silva et al., 2024), hematological neoplasms (Phillips et al., 2022), and sepsis in calves (Uztimür, 2024). The study suggests that PDW/PLT may be useful for monitoring localized or systemic inflammatory responses in veterinary species, but further study is needed to determine its clinical relevance.

#### 3.2.5 Platelet large cell ratio (PLCR)

PLCR stands for Platelet Large Cell Ratio, which reflects the proportion of large platelets in circulation, typically younger and more hemostatically active. Large platelets are often observed when the bone marrow releases newly formed platelets into the bloodstream during conditions with increased platelet turnover, such as acute inflammation, immune-mediated thrombocytopenia, or cancerous processes (Scherlinger et al., 2023; Scridon, 2022; Falanga et al., 2025). Studies in veterinary medicine have shown that PLCR varies according to the state of the disease. It has been reported that dogs with thrombocytopenia have elevated PLCR values, indicating an increased presence of large, young platelets during compensatory responses (Souza, 2022).

A significant difference in PLCR was observed in dogs with oropharyngeal tumors when compared to healthy controls or dogs with periodontal inflammation, indicating a marker of systemic or localized pathological platelet activation (Rejec et al., 2017). In dogs with hematologic neoplasia, PLCR has also been shown to increase when platelets are abnormally produced or turned over (Phillips et al., 2022). PLCR could serve as an effective biomarker in detecting increased platelet activation or turnover in inflammatory, immune-mediated, and neoplastic conditions, although standardized reference ranges and diagnostic utility need to be determined.

### 3.3 Red cell-related ratios

#### 3.3.1 Red blood cell distribution width-to-platelet ratio (RDW/PLT or RPR)

Red blood cell distribution width (RDW), calculated as the standard deviation of the mean corpuscular volume (MCV) multiplied by 100, helps characterize human and animal anemia. A high RDW value indicates increased heterogeneity and variation in the size of RBCs. Various metabolic abnormalities may contribute to an increased RDW, including inflammation, oxidative stress, poor nutritional status, dyslipidemia, hypertension, erythrocyte fragmentation, and changes in erythropoietin function (Yuyun et al., 2019; Kucera et al., 2015; Afzal, 2025). Inflammation has been shown to inhibit RBC maturation and shorten their lives (Kyriacou & Shibeeb, 2025). RDW values may be elevated in dogs, cats, and horses with systemic diseases, which include cardiomyopathies, infections, etc. (Scalco et al., 2023b; Hu et al., 2020; Martin et al., 2019). RDW/PLT (also known as RPR) integrates erythrocyte sizing variability into platelet count and is increasingly being used as a biomarker of inflammation and disease severity in veterinary patients. The RPR is characterized by erythrocyte anisocytosis as well as thrombocytopenia, commonly associated with systemic inflammation and sepsis. A higher RPR was observed in dogs with worse clinical outcomes due to canine parvoviral infection (Varlik et al., 2026). This was likely reflecting increased inflammatory response, increased bone marrow stress, and consumptive coagulopathy. High RPR values indicate poorer prognoses, making them useful markers for identifying high-risk septic patients (Varlik et al., 2026).

The RPR of dogs who had myxomatous mitral valve disease in various stages decreased significantly when compared with healthy controls, which was associated with markers of oxidative stress and systemic inflammation. The results suggest that lower RPR levels are associated with chronic cardiovascular dysfunction and hemodynamic stress (Kocaturk et al., 2024). In dogs with heart failure, RPR appeared to be more sensitive when the cut-off value is set to 0.057 than with NLR, making it better to predict disease severity, both in symptomatic and asymptomatic dogs (Kocaturk et al., 2024). Among dogs with myxomatous mitral valve disease, it was found to be an independent prognostic factor for poor outcomes (Guglielmini et al., 2021).

Conversely, acute inflammatory conditions such as hemorrhagic enteritis in dogs significantly increased RPR, emphasizing its responsiveness to acute intestinal inflammation and rapid hematological changes (Arslan et al., 2017). It was found that dogs with hepatitis caused by different etiologies, including liver failure, had high levels of RDW and RPR (Çolakoğlu et al., 2025). The RPR is an effective indicator of liver fibrosis and cirrhosis caused by severe hepatitis (Taefi et al., 2015).

Additionally, researchers have investigated neonatal foals, revealing that at-risk or septic foals had higher RPR values than healthy foals within the first 24 h of life, indicating that perinatal complications and sepsis were causing early hematologic and inflammatory responses (Scalco et al., 2023a, 2023b). Inflammatory processes increase RDW, since the release of mediators such as tumor necrosis factor, IL-6, and IL-1 is proposed to suppress both erythropoiesis and RBC maturation, as well as decrease their half-life (Jo et al., 2013). As part of the acute phase inflammatory response, the mononuclear phagocyte system is also activated, which increases the destruction of RBCs (Richards et al., 2016). It is also known that erythrocyte size is more variable during acute hypoxic insults during sepsis (Haase, 2010). A well-established role of platelets in sepsis is to mitigate inflammation, modulate immune responses, and assist in hemostasis and thrombosis (Oncel et al., 2012). When injured or infected, platelets are rapidly mobilized and activated. Critically ill foals are commonly seen with coagulopathy and thrombocytopenia due to consumption (Scalco et al., 2023a; Oncel et al., 2012). Further, RPR increased in adult horses with piroplasmosis and showed predictive value for infection status when molecular diagnostics are not immediately available (Duaso et al., 2025).

Across animal species, these studies demonstrate that RPR can increase or decrease based on disease nature and chronicity. There remain many questions to be answered, both in terms of establishing species-specific reference intervals and defining normal and pathological thresholds.

#### 3.3.2 Red blood cell distribution width-to-lymphocyte ratio (RDW/LYM)

This hematologic index combines two biologically meaningful markers, RDW/LYM, as a derived hematologic index. A higher RDW indicates systemic inflammation, oxidative stress, and impaired erythropoiesis, and a reduced lymphocyte count indicates an inflammatory burden and immunosuppression related to stress. Combined, these parameters maximize the indication of an inflammatory imbalance better than either one alone (Hsieh et al., 2024; Tezol et al., 2020). There have been studies conducted in veterinary medicine that utilize RDW/LYM primarily for conditions characterized by widespread inflammation or hepatic involvement. An increase in RDW/LYM was observed in dogs with primary cholangitis or cholangiohepatitis and was proposed as a potentially useful clinical parameter (Çolakoğlu et al., 2025). The RDW of patients with advanced-stage cirrhosis and progressed hepatic disease was significantly higher, lymphocyte counts were significantly lower, and the RDW/LYM ratio was significantly higher when compared to patients with early-stage cirrhosis (Çolakoğlu et al., 2025; Meng et al., 2018). Moreover, liver biopsy confirmed the disease staging in the study (Meng et al., 2018). The RDW/LYM can be a manifestation of oxidative stress, disordered erythropoiesis, and lymphopenia, which is associated with septic shock and immune suppression. In severely affected dogs with canine parvovirus, which was associated with systemic inflammation, RDW/LYM levels were significantly elevated (Varlik et al., 2026). Based on its strong association with disease severity, RDW/LYM may be useful for identifying early risk factors (Varlik et al., 2026).

RDW/LYM indices and RDW-derived indices have also been examined for heart diseases in dogs, in particular myxomatous mitral valve disease and heart failure, conditions in which inflammation and oxidative stress contribute to disease progression (Kocaturk et al., 2024). Furthermore, RDW combined with leukocyte-based ratios is a prognostic indicator for severe inflammatory conditions like acute pancreatitis, supporting the inclusion of lymphocyte counts in composite indices (Johnson et al., 2023).

#### 3.3.3 Red blood cell distribution width-to-mean platelet volume ratio (RDW/MPV)

The RDW/MPV ratio integrates two biologically distinct and inflammation-responsive pathways into a composite hematologic index: Increased RDW reflects oxidative stress, disordered erythropoiesis, and systemic inflammation, while elevated MPV indicates platelet activation and megakaryocytic response to inflammation (Cakir & Tayman, 2025). By assessing RDW and MPV together, we can measure erythrocytic heterogeneity and platelet activation or turnover. Developing such an index could assist us in understanding disease pathophysiology, especially in conditions involving inflammation, oxidative stress, or dysregulation of bone marrow (C Li et al., 2024; Karagoz et al., 2017). It has been evaluated in veterinary medicine in conditions associated with systemic inflammation or cardiovascular stress. RDW/MPV levels were greater in nonsurvivors of sepsis associated with canine parvoviral infection, and these ratios correlated with other CBC-derived ratios (Varlik et al., 2026). In addition, dogs with myxomatous mitral valve disease and progressive stages of heart failure also displayed altered RDW and MPV to correlate with inflammatory and oxidative status (Kocaturk et al., 2024).

#### 3.3.4 Red blood cell distribution width-to-total serum calcium ratio (RDW/Ca)

Ratio of RDW to Total Serum Calcium has been investigated and proposed as a prognostic indicator only in patients with acute pancreatitis in both humans and dogs (Han et al., 2022; Jafari et al., 2025; Guglielmini et al., 2022). RDW and serum calcium alone can be used to predict acute pancreatitis severity; however, they are not highly sensitive or specific (Han et al., 2022). An association between hypocalcemia and acute pancreatitis severity appears to be caused by calcium saponification, and hypocalcemia can be a concern in dogs with acute pancreatitis (Guglielmini et al., 2022; Holowaychuk, 2013). Human RDW/Ca ratios are excellent indicators of acute pancreatitis severity and mortality (Han et al., 2022). The RDW/Ca of dogs with acute pancreatitis was significantly higher in nonsurvivors than in survivors, suggesting that hypocalcemia may be a concern in these dogs, and RDWs > 12.7 % were effective as a predictor of short-term mortality (Guglielmini et al., 2022).

#### 3.3.5 Hemoglobin-to-red cell distribution width ratio (Hb/RDW)

The hemoglobin to RDW ratio combines hemoglobin concentration and heterogeneity of red blood cells. Human studies demonstrate that Hb/RDW predicts inflammation, disease severity, and prognosis in conditions including cardiovascular disease, critical illness, and cancer (Uğuz et al., 2024; Chen et al., 2020). Generally, lower Hb/RDW ratios are associated with anemia and anisocytosis, both of which are associated with poorer outcomes (Xu et al., 2025b). There are no studies specifically calculating or validating the Hb/RDW ratio in animals, despite Hb and RDW being routinely reported in complete blood counts across species. There is a clear gap in the veterinary patient population, suggesting Hb/RDW might be a useful marker for integrating anemia severity and erythrocyte heterogeneity in clinical and prognostic assessments.

### 3.4 Composite and novel indices

#### 3.4.1 Systemic immune-inflammation index (SII) = (Platelets × neutrophils) / lymphocytes

The systemic immune/inflammation index (SII) has gained attention as one of the key markers measuring the severity of inflammatory responses in animals (Sindhu et al., 2025). Further studies on immune cell dynamics as a measure of chronic inflammation led to the development of the SII, which is calculated by multiplying the number of neutrophils and platelets by the number of lymphocytes (Tort et al., 2023; Satis, 2021). There can be a negative correlation between this index and the prognosis of solid tumors, but it can also predict autoimmune outcomes, such as rheumatoid arthritis and heart disease (Satis, 2021). Composite ratios indicate systemic inflammation and can provide valuable insight into the patient's overall health status (Ji et al., 2021). Several studies have suggested SII can serve as a marker of chronic inflammation, demonstrating decreased lymphocytes and increased neutrophils and platelets (Ye et al., 2023).

The SII has been tested in dogs under various conditions in conjunction with other CBC-derived inflammatory indices. Increased SII values in critically ill patients are associated with severe systemic inflammation and worse outcomes (DeProspero et al., 2023; Durán-Galea et al., 2024; Neumann, 2021; Cristóbal et al., 2022). Furthermore, using inflammation indices, such as SII, clinicians may be able to initiate therapeutic interventions sooner, thereby improving clinical outcomes (DeProspero et al., 2023; Pierini et al., 2020; Cristóbal et al., 2022). Hematological abnormalities such as neutrophilia (27–94 %), lymphopenia (2–29 %), and thrombocytopenia (14–73 %) are common in canine leptospirosis and may significantly affect SII values (Sykes et al., 2011). In dogs with canine leptospirosis, SII was higher than in healthy dogs, but it was not associated with survival, suggesting it may be more useful for morbidity assessment than for mortality prediction (Durán-Galea et al., 2024).

During canine monocytic ehrlichiosis, increased SII reflects systemic inflammatory responses, which may allow physicians to monitor disease progression and severity (Erdogan et al., 2025). When it comes to other systemic infections, such as viral and bacterial infections, SII has demonstrated greater sensitivity than single-parameter indices such as NLR or PLR (Sindhu et al., 2025). Dogs with multiple organ dysfunction due to systemic inflammatory response syndrome showed even greater SII values (Sindhu et al., 2025). The SII has also been used to track response to mesenchymal stem cell therapy in dogs with chronic inflammatory enteropathy, showing that a decline in SII correlates with better clinical outcomes and reduced inflammation (Cristóbal et al., 2022). The SII was used to monitor postoperative inflammatory processes in dogs undergoing open versus laparoscopic ovariectomy, showing a significant increase after surgery (Espadas-González et al., 2023).

Veterinary oncology has also been proven to benefit from SII. In canine oral melanoma treated with experimental immunotherapy alone or in combination with metronomic chemotherapy, high SII values were correlated with shorter overall survival and poorer therapeutic response, supporting its role as a reliable prognostic biomarker (Garcia et al., 2022). It has been shown that increased SII is associated with tumor-induced inflammation and immune suppression, contributing to clinical decision-making and outcome prediction (Sindhu et al., 2025).

Obesity is one of the most prevalent metabolic disorders in pet animals, and it has been associated with chronic inflammation. A study demonstrated an association between SII and CRP in obese dogs (Tuna & Ulutaş, 2024). Diabetes mellitus in dogs is also associated with a marked increase in SII compared to healthy dogs with no diabetes, which is consistent with autoimmune dysregulation (Vaitaitis et al., 2024). Meanwhile, in feline arterial thromboembolism, elevated SII values (>441), together with other inflammatory indices, have been associated with poor prognosis and reduced survival rates (Esin & Uzun, 2025).

Research conducted on horses found that those with elevated acute-phase proteins had higher SII values than those with non-inflamed controls, demonstrating the validity of this measure over traditional CBC parameters and leukocyte ratios in equine medicine (Friend et al., 2025). During intense training, SII fluctuates dynamically in show-jumping horses in response to exercise-induced stress and recovery, reflecting transient immuno-inflammatory changes (Alves-Junior & Ferreira, 2025). Similarly, SII provided insight into physiological stress and recovery patterns in endurance horses (de Siqueira & Fernandes, 2024). Further, in neonatal foals with acute infections, monitoring SII dynamically allowed real-time assessment of inflammation progression and treatment response, suggesting its use as a sensitive early biomarker in critical care (Alves-Junior). It was found that neonatal calves with diarrhea and systemic inflammatory response syndrome had greater SII than controls, suggesting SII can indicate systemic inflammation (Aydın & Yıldırım, 2024).

#### 3.4.2 Chronic inflammation index (CII) = (Platelets × monocytes) / lymphocytes

According to one study, the SII formula could be modified to include monocytes rather than neutrophils (Vaitaitis et al., 2024). The chronic inflammation index (CII) is calculated by multiplying the number of monocytes and platelets by the number of lymphocytes. A significantly higher CII was found in dogs with canine diabetes mellitus, with a narrower range than in SII (Vaitaitis et al., 2024). The number of monocytes representing innate immune cells was elevated in diabetic dogs compared with control dogs. Despite increased neutrophil count in diabetes mellitus, the difference between diabetic and healthy neutrophil counts was not meaningful (Vaitaitis et al., 2024). Diabetes occurs when monocytes attack islets and release inflammatory cytokines, causing autoimmune destruction of pancreatic islets (Jansen et al., 1994).

#### 3.4.3 Systemic inflammation response index (SIRI) = (Neutrophils × monocytes) / lymphocytes

Systemic inflammation response index (SIRI) has recently emerged as one of the most promising hematological biomarkers for assessing systemic inflammation across a wide range of animal species. A significant increase in SIRI was observed in dogs with Canine Monocytic Ehrlichiosis presenting with systemic inflammatory response syndrome, demonstrating SIRI's potential as an indicator of acute inflammatory states (Erdogan et al., 2025). As well, SIRI values were elevated in neonatal calves with diarrhea and SIRS compared with healthy controls, indicating activation of innate immunity and a pro-inflammatory response imbalance (Aydın & Yıldırım, 2024). There is evidence that high SIRI levels in cats with feline arterial thromboembolism correlate with a worse prognosis and a shorter survival time, suggesting its potential use in risk stratification and outcome prediction (Esin & Uzun, 2025). Further, SIRI levels were significantly elevated in cats suffering from advanced cardiomyopathy, indicating it is a potential marker of chronic systemic inflammation in cardiac disease (Krofič Žel et al., 2025).

Equine medicine has investigated the SIRI as an integrative biomarker reflecting the balance between innate and adaptive immunity. The SIRI is shown to increase in horses with systemic inflammation, for a more sensitive measurement than single leukocyte ratios alone (Friend et al., 2025). During exercise-induced stress and recovery in show-jumping and endurance horses, SIRI dynamically fluctuates, capturing transient immuno-inflammatory changes that can influence performance (Alves-Junior & Ferreira, 2025; de Siqueira & Fernandes, 2024). Moreover, SIRI detected critical illness and guided therapeutic interventions in neonatal foals with acute infections, highlighting its potential as an early biomarker for critical illness (Alves-Junior).

#### 3.4.4 Aggregate index of systemic inflammation (AISI) = (Neutrophils × monocytes × platelets) / lymphocytes

A composite value based on neutrophils, monocytes, platelets, and lymphocytes is calculated using the AISI formula. This is based on the concept that systemic inflammation is characterized by simultaneous neutrophilia, monocytosis, thrombocytosis, and relative lymphopenia. It is possible to detect inflammation quickly and sensitively by multiplying proinflammatory cell lines and dividing them by lymphocytes. The use of AISI in veterinary medicine has increased in recent years. The AISI value is an accurate prognostic and diagnostic tool for feline arterial thromboembolism, as shown by the lower survival rate and worse prognosis of cats with high values (Esin & Uzun, 2025). There have also been studies of AISI in horses as part of CBC inflammatory index-derived studies, including a study of inflammatory responses in clinically affected horses (Friend et al., 2025), an assessment of immune-integrative biomarkers in show-jumping horses under athletic stress (Alves-Junior & Ferreira, 2025), and a dynamic assessment of neonatal infections (Alves-Junior). Preliminary results indicate that AISI has the potential to serve as an affordable and useful biomarker for inflammatory conditions across various animal species.

## 4 Discussion

Diagnostic and prognostic hematological ratios in veterinary medicine have been examined extensively. Given their availability and the need to minimize diagnostic costs, their calculation was considered, and their results were highly correlated with those of other reliable methods. For instance, the NLR and MLR showed a positive correlation with SAA and globulins, as well as a negative correlation with albumin and AGR. Cats with elevated levels of SAA and globulins and low albumin and AGR had higher NLR and MLR. Those cats with left-shift neutrophils, toxic neutrophils, and reactive lymphocytes exhibited higher NLR, MLR, and PLR (Donato et al., 2023). Calculating ratios should always be done using absolute blood cell counts. Since blood cell absolute counts are expressed as hematological ratios, multiple confounding factors, such as dehydration, are not included in the equation (Atata et al., 2018). As a result, comparative and relative ratios can be helpful rather than absolute metrics.

**Fig. 1:**
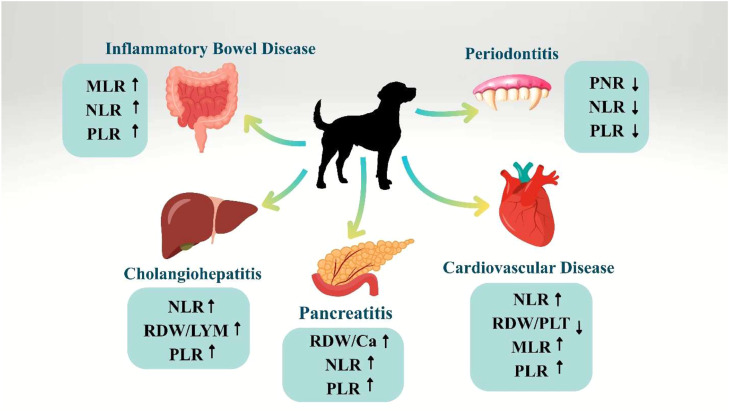
Inflammatory conditions in dogs are listed along with the hematological ratios used in studies and reported to be effective in diagnosing them, along with their decrease or increase. Combining these ratios can provide valuable information alongside other diagnostic tools. Abbreviations: NLR, neutrophil to lymphocyte ratio; MLR, monocyte to lymphocyte ratio; PLR, platelet to lymphocyte ratio; PNR, platelet to neutrophil ratio; RDW/LYM, red blood cell distribution width to lymphocyte ratio; RDW/PLT, red blood cell distribution width to platelet Ratio.

**Fig. 2:**
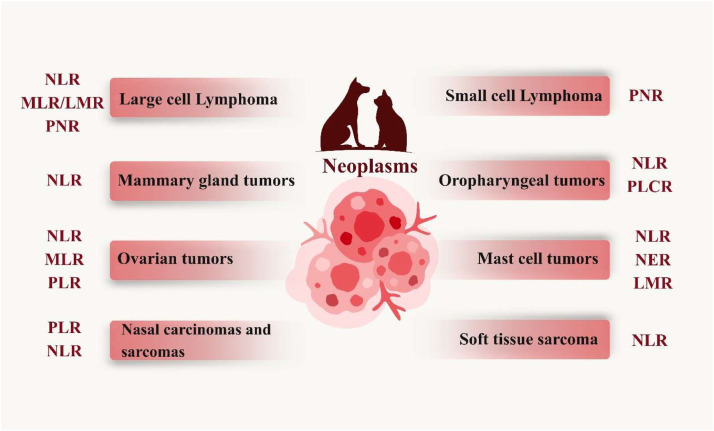
The performance of certain hematological ratios for a specific number of prevalent malignancies in dogs and cats has been shown to be superior in terms of diagnosis, staging, and prognosis. It is beneficial to incorporate these ratios into the profile of animals with cancer. Abbreviations: NLR, neutrophil to lymphocyte ratio; MLR, monocyte to lymphocyte ratio; LMR, lymphocyte to monocyte ratio; PLR, platelet to lymphocyte ratio; PNR, platelet to neutrophil ratio; NER, neutrophil to eosinophil ratio; PLCR, platelet large cell ratio.

Specific ratios can be calculated for each disease, based on the most prominent symptoms and hematological changes, which are most affected by the disease symptoms and least affected by other influential factors. As an example, dogs with ovarian tumors show mild anemia, neutrophilia, thrombocytopenia, and lymphopenia. Using PLR, NLR, and MLR to predict ovarian tumors in dogs is highly efficient (Uğur et al., 2025). It is very critical that when assessing disease status or prognosis, one does not rely on just one hematological ratio. The alternative method is to evaluate multiple hematological ratios, preferably those related to the cell lines with the most pronounced changes, in conjunction with other common diagnostic procedures. Multiple hematological ratios can be combined in diagnostic or prognostic panels to provide comprehensive information. Leukocyte, erythrocyte, and platelet ratios could be integrated to assess systemic inflammation, bone marrow responses, and disease progression effectively (Fig. 1). Inflammatory and neoplastic processes can be evaluated more comprehensively by monitoring NLR alongside RPR or PLR than by using single ratios (Argın et al., 2025) (Fig. 2).

These ratios have a slight disadvantage in terms of sensitivity and specificity because they have a lower discrimination ability (Silva et al., 2024). Thus, hematological tests cannot replace all other tests. Hematological ratios are recommended as a rapid, feasible, and complementary diagnostic approach, in combination with other diagnostic approaches based on clinical symptoms. For instance, it has been shown that PNR, NLR, and PLR can detect canine periodontal disease, although a thorough dental assessment, including an intraoral radiograph, is key to making an accurate diagnosis (Silva et al., 2024). Moreover, hematological ratios may be useful to veterinarians when assessing the oral cavity, particularly when sedation is difficult or not feasible. Routine blood tests and analysis of the ratios can demonstrate the systemic effects of periodontal diseases in these situations.

Exogenous or endogenous corticosteroids, such as those produced by stress, can cause a stress leukogram, which can be accompanied by neutrophilia, lymphopenia, and monocytosis in some species. Consequently, it can affect the ratios that these cells are based on, although typically the increase in indicators such as NLR is not as pronounced as in actual inflammatory conditions. As an example, one study suggests that NLR elevation and, to a lesser extent, LMR decrease might be stress-related in cats, likely due to a corticosteroid-induced induction of neutrophilia, lymphopenia, and possibly monocytosis (Tsouloufi et al., 2021). As cats have a greater proportion of neutrophils in the marginal pool than other species, neutrophil demarginating is the earliest mechanism responsible for the increase of mature circulating neutrophils (Rossi, 2023). Cytokines stimulate the release of neuroendocrine molecules associated with inflammation, which affect both leukocyte populations and acute phase proteins (Paltrinieri, 2008). These ratios must be correlated with other sensitive biomarkers of inflammation, such as acute-phase proteins like SAA and CRP, proinflammatory cytokines like IL-6, and stress, since inflammation and corticosteroid-mediated stress often coexist (Tsouloufi et al., 2021). It must be noted, however, that stress itself can be aggravating in severe conditions, such as septicemia (Li & Rong, 2025). Additionally, in conditions like hyperadrenocorticism and hypoadrenocorticism, attributed directly to changes in corticosteroids, NLR and PLR ratios have been demonstrated to be useful (Zeugswetter & Schwendenwein, 2014; Yun et al., 2024).

In spite of extensive research on hematological ratios in domestic animal species, different cut-offs and contradictory numbers can be confusing. The hematological ratios have been studied in several veterinary studies specifically or semi-specifically for establishing reference values and suggest cut-offs (Donato et al., 2023; Cristóbal et al., 2022; Navarro et al., 2025). However, it is important to note that results vary widely between studies, and the methodology (sample, analyzer, age/breed, Reference Interval calculation algorithm) has varied, so it is important to be cautious when interpreting the results.

There is a recent study in healthy dogs that has established reference intervals for several hematological ratios, establishing the first standardized baselines. A NLR range of 1.3 to 7.1, a MLR range of 0.1 to 0.3, and a PLR range of 40.4 to 271.1 were recorded in the population of normal dogs, whereas the SII showed a wider range, with a reference interval of 224.6 to 2191.7 (Navarro et al., 2025). NLR showed a statistically significant difference by sex, with males having a higher ratio than females, and dogs of different ages having significantly different NLRs. There were also higher ratios of PLR and SII in senior dogs when compared to adults and juveniles (Navarro et al., 2025). The reference range has not been studied exclusively in cats, but a clear cut-off has been reported for discerning inflammatory conditions according to some of the hematological ratios. Among cats without any changes in parameters indicative of inflammation and appearing healthy, the upper limits for NLR, MLR, and PLR were 11.25, 0.42, and 528.3 based on a study (Donato et al., 2023).

Recently, a population-based study examined horses with normal acute phase protein concentrations (SAA and haptoglobin) as non-inflamed controls to establish non-inflamed reference intervals for CBC parameters and composite inflammatory indices, including SII, SIRI, and AISI (Friend et al., 2025). A normal value of 157.7 has been reported for SII (45.5–348.5), 0.56 for SIRI (0.23–1.22), and 57.3 for AISI (13.6–137.6) in horses (Friend et al., 2025). Research on cattle and other ruminants has not provided evidence of the determination of normal ranges for hematological ratios. Reference intervals for other hematological ratios have not been studied specifically. Clinical studies using healthy control groups can also provide supporting reference data. In research evaluating inflammatory biomarkers in cardiovascular, gastrointestinal, and neoplastic conditions, practical upper limits were defined for these ratios (Gavazza et al., 2024; Jung & Kim, 2023; DeProspero et al., 2023; Marchesi et al., 2024; Cristóbal et al., 2022). Despite not being designed to generate formal reference intervals, the control cohorts contribute to a better understanding of baseline patterns and emphasize the need for standardized, analyzer-specific reference intervals. The presence of abnormal ratios in animals without clinical symptoms does not always indicate a special disease. However, it can indicate the need for complementary tests and physical examinations.

Composite ratios capture changes in inflammation patterns more accurately and delicately because they are calculated as multiples of changes. This makes them more efficient in addressing small and subtle changes when the manifestations of systemic alterations aren't as obvious (de Siqueira & Fernandes, 2024). There was consistency in the value of SII, SIRI, and AISI in identifying inflammation in equids. These inflammatory indices had significantly higher median values in inflamed horses and superior predictive value than other blood cell parameters and cell ratios (Friend et al., 2025). Composite ratios have garnered more attention in equine medicine, perhaps because horses' inflammatory responses are less severe than those of small pets like dogs and cats (Friend et al., 2025; Alves-Junior & Ferreira, 2025). Large animals, such as horses and cattle, exhibit a slower, milder bone marrow response to infection and inflammation than dogs and cats (Lester et al., 2015). As platelets, granulocytes, and monocytes are seen as a composite and are deviated by lymphocytes, ratios such as SII, SIRI, and AISI can yield more accurate information than other blood ratios, which may give contradictory results (Erdogan et al., 2025; Friend et al., 2025). The use of hematological ratios in equine diseases such as colic and laminitis remains a gap in the literature that could be addressed in future studies. Several critical steps must be taken to enable early diagnosis of these diseases in order to identify animals at risk and to provide treatment before severe clinical signs emerge, and blood cell ratios may provide some insight into these diseases (Moore et al., 1981; Purnama et al., 2022).

Hematological ratios have been used for cattle and ruminants only very rarely, and little research has been done on this topic (Aktaş et al., 2024; Aktas et al., 2023; Gupta et al., 2025). Despite the fact that bone marrow responses and changes in cell count in bovine blood occur slowly and don't have much value in detecting mild inflammation, they are useful in detecting some diseases, such as respiratory diseases of calves (Aktaş et al., 2024), and theileriosis (Aktas et al., 2023; Gupta et al., 2025). NLR and PLR levels were elevated in 57.89 % and 42.10 % of cattle infected with *Theileria annulata*, respectively, while 31.57 % displayed elevated levels of both NLR and PLR (Gupta et al., 2025). A systemic inflammation was reported in the majority of cases of bovine theileriosis, which influenced their outcomes. The presence of severe leukocyte changes in cattle is usually reported in situations such as severe infections, septicemia, and diseases such as bovine leukemia. Ruminants exhibit different patterns of blood cell changes from other domestic animals, indicating the necessity for standardization and setting appropriate ratios based on the animals' individual circumstances (Johns & Heller, 2021; Kessell, 2015).

Veterinary medicine faces challenges with regard to hematological ratios because of the discrepancy between the cell counter devices used to analyze blood from various species (Voigt & Swist, 2011). For example, due to differences in platelet size in cats and the formation of platelet clumps in animals like cattle and cats, the number and volume of platelets can vary widely, leading to inconsistent results across repeated studies and low reproducibility when using different devices (Schaefer, 2022; Wasserkrug-Naor, 2022). Pseudo-thrombocytopenia often occurs in normal cats because they tend to develop platelet aggregations (Zanfagnini et al., 2021). There is therefore a need for a comprehensive standardization system, especially in the case of platelets, as they are more sensitive to variations than other cell lines (Wasserkrug-Naor, 2022; Cook et al., 2016).

Breed differences in animals, particularly in dogs, can result in significant variations in hematological ratios. For instance, Cavalier King Charles Spaniels generally exhibit lower platelet counts than other breeds and may also exhibit giant platelets (Bertazzolo et al., 2007). Red blood cell size and MCV also differ among certain breeds. Asian breeds such as Akita, Chow, and Shar Pei possess smaller red blood cells, whereas breeds like Poodles may demonstrate macrocytosis (Edition). Consequently, breed serves as a confounding factor in the assessment of hematological ratios in dogs (Gavazza et al., 2012). Separate reference ranges should be established for these breeds, as values proposed in general studies may not be applicable. These breeds are sometimes excluded from population studies or complicate the interpretation of reference intervals (Misbach et al., 2014). In such cases, alternative hematological ratios, including those related to leukocytes, may be utilized as in other canine populations. Using a systematic diagnostic protocol in domestic animals incorporating factors that can be analyzed quickly, such as blood test indicators and ratios, along with clinical examination and other diagnostic techniques, enables the reduction of diagnostic costs and reduced consultation time when assessing domestic animal diseases (Jia et al., 2020). In addition, conducting a comprehensive meta-analysis study in which numbers and data are pooled from several studies conducted on various animal species would provide an overview of how these ratios will be applied in clinical as well as practical settings, as well as the method of assessing outcomes. A comprehensive standard framework for their application in the clinical and practical setting may be necessary at some point in the future in order to reduce the possibility of inconsistent results.

## 5 Conclusion

Veterinary medicine has paid more attention to some hematological ratios, including NLR, MLR, LMR, PLR, and RPR. Increases and decreases in each of these ratios can have different interpretations, so it is best to use these ratios together in conjunction with one another for the diagnosis and prognosis of diseases, as they provide a more complete picture of individual disease processes. Due to the importance of blood cell dynamics in determining the adequacy of an animal's immune system, the severity of the damage inflicted, the chronicity and nature of the illness, the effectiveness of treatment, survival rates, and outcomes, these ratios have gained attention in inflammatory conditions, neoplasms, degenerative diseases, and autoimmune disorders. An increase in these ratios is more effective than an increase in absolute blood parameters, and they often change along with other inflammatory factors, such as acute-phase proteins. It is necessary to note that hematological ratios alone may not be sufficient, but when combined with physical examinations, signalments, and other clinical and paraclinical tests, they can provide efficient, rapid, and cost-effective diagnostic information. The majority of research has been conducted on small animals, mostly dogs and cats. Composite ratios have recently shown promising results in large animal medicine, especially for horses. Meta-analysis can be used in the future to integrate research results into a unified protocol for the diagnosis of the pathologies occurring in animal species, such as tumors, infections, cardiovascular conditions, etc.

## Funding

No funding received.

## Ethical approval

All applicable international, national, and/or institutional guidelines were followed.

## CRediT authorship contribution statement

**Narges Lotfalizadeh:** Writing – review & editing, Writing – original draft, Validation, Investigation. **Saeed Nazifi:** Writing – review & editing, Writing – original draft, Validation, Supervision, Methodology, Investigation, Conceptualization.

## Declaration of competing interest

The authors declare no conflict of interest.

## Data Availability

The datasets generated during and/or analyzed during the current study are available from the corresponding author upon reasonable request.
